# A Highly Reliable and Cost-Efficient Multi-Sensor System for Land Vehicle Positioning

**DOI:** 10.3390/s16060755

**Published:** 2016-05-25

**Authors:** Xu Li, Qimin Xu, Bin Li, Xianghui Song

**Affiliations:** 1 School of Instrument Science and Engineering, Southeast University, Nanjing 210096, China; jimmy.xqm@gmail.com; 2 Key Laboratory of Technology on Intelligent Transportation Systems Ministry of Transport, Research Institute of Highway Ministry of Transport, Beijing 100088, China; libin@itsc.cn (B.L.); sxh@itsc.cn (X.S.)

**Keywords:** vehicle positioning, distributed-dual-H∞ filtering, reduced inertial sensor system, generalized regression neural network

## Abstract

In this paper, we propose a novel positioning solution for land vehicles which is highly reliable and cost-efficient. The proposed positioning system fuses information from the MEMS-based reduced inertial sensor system (RISS) which consists of one vertical gyroscope and two horizontal accelerometers, low-cost GPS, and supplementary sensors and sources. First, pitch and roll angle are accurately estimated based on a vehicle kinematic model. Meanwhile, the negative effect of the uncertain nonlinear drift of MEMS inertial sensors is eliminated by an H∞ filter. Further, a distributed-dual-H∞ filtering (DDHF) mechanism is adopted to address the uncertain nonlinear drift of the MEMS-RISS and make full use of the supplementary sensors and sources. The DDHF is composed of a main H∞ filter (MHF) and an auxiliary H∞ filter (AHF). Finally, a generalized regression neural network (GRNN) module with good approximation capability is specially designed for the MEMS-RISS. A hybrid methodology which combines the GRNN module and the AHF is utilized to compensate for RISS position errors during GPS outages. To verify the effectiveness of the proposed solution, road-test experiments with various scenarios were performed. The experimental results illustrate that the proposed system can achieve accurate and reliable positioning for land vehicles.

## 1. Introduction

The most widespread land vehicle positioning systems are those which integrate a Global Positioning System (GPS) and an Inertial Navigation System (INS). Both systems are complementary, and their integration provides superior performance to either of them operating alone [1,2,3,4]. For instance, GPS generally provides satisfactory performance in open fields and a GPS-derived position can be used to improve long-term accuracy of INS. However, in urban canyons, tunnels, and other GPS-denied environments, the GPS satellite signal is blocked, and there is an interruption in the positioning information provision [5]. *Vice-versa*, INS is a self-contained system which is not affected by external disturbances. However, its accuracy deteriorates in the long term due to the integration of inertial sensor errors. INS can also be used to bridge GPS outages and reduce the search domain required for detecting and correcting GPS cycle slips [6].

Literature review shows that the filters of INS/GPS integration are typically some forms of Kalman filter (KF), including conventional KF, Extended KF, and Unscented KF [7,8,9,10]. The major limitation related to the utilization of the KFs is the necessity to have predefined accurate models for each sensor error [3,5,11,12]. The filter performance is highly dependent on the accuracy of sensor error models. For high-quality INS, the INS sensor errors can be accurately modeled and estimated, and then the KFs provide precise corrections for INS.

However, the high cost and government regulations prevent the wider inclusion of high-quality INS to augment GPS as a commercial system for land vehicle positioning [8]. The progress in micro-electro-mechanical system (MEMS) technology has led to the production of low cost inertial measurement units (IMU) with three sets of accelerometers and gyroscopes placed along three mutually orthogonal directions. In order to further lower the cost, research efforts have recently been made to investigate the applicability of reduced inertial sensor systems (RISS), especially MEMS-based RISS (MEMS-RISS). Since the price of the gyroscopes mostly contributes to the overall cost of an IMU [13], only one vertical gyroscope is usually retained in RISS. Different numbers of accelerometers are used depending on different applications. The application of RISS can be classified into two categories: (1) a 2D positioning solution where GPS is integrated with only one single-axis gyroscope and an odometer [14,15,16]. With the assumption that the vehicle mostly stays in the horizontal plane, the vehicle speed obtained from the odometer is used together with the yaw angle information obtained from the gyroscope to determine the velocities along the east and north directions. Consequently, the vehicle’s longitude and latitude are determined; (2) a 3D positioning solution where RISS involving single-axis gyroscope and two-axis accelerometers is integrated with GPS [5,17]. A general method for this integration is to replace the omitted gyroscopes and accelerometer with corresponding pseudo-signals, namely zero for gyroscopes and standard gravity for vertical accelerometer, to perform INS mechanization. Usually, other sensors such as odometers and monocular cameras [18] can be introduced to enhance the positioning performance. Besides, pitch and roll angle can be calculated from the two accelerometers mounted along the longitudinal and lateral axes of the vehicle. However, one of the inadequacies is that the angles are directly calculated without filtering.

The major limitation related to the MEMS inertial sensor is the high uncertainty of sensor errors in its measurements [19]. However, the uncertain inertial sensor errors have not been thoroughly considered in both categories of positioning solution above. In all the MEMS inertial sensor errors, initial errors and scale factor errors can be reduced through several ground platform tests. The residual errors, termed noises or drifts, are usually expressed using stochastic models such as Gauss-Markov models and autoregressive models [7,8] in KFs solutions. However, these models only provide an approximate description about the behavior of inertial sensor errors. As long as GPS is available, despite the modeling errors caused by uncertain nonlinear drift, the RISS errors can be corrected by GPS position and velocity measurements. Nevertheless, during GPS outages, the modeling errors will lead to the performance degradation of KFs [20,21,22], and the RISS errors cannot be corrected.

Alternatively, the H∞ filter has been developed as a robust filtering method. The H∞ filter does not make any assumptions about the error’s characteristics, but regards it as energy-bounded signal [23,24,25,26,27]. In other words, the H∞ filter does not need to have predefined error models. Thus, the H∞ filter is essentially immune to the uncertain nonlinear drift of MEMS inertial sensors. This advantage motivates the use of the H∞ filter in this paper.

In the last few years, artificial neural network (ANN) approaches, such as the Radial Basis Function (RBF) neural networks [28,29,30] and the Adaptive Neuron-Fuzzy Inference System (ANFIS) [8,31], have been developed to enhance GPS/INS integration for its improved ability to handle the problem of lacking systemized set of functions that can correctly characterize the relationship between inputs and outputs. Usually, the inputs of ANN models are INS and/or IMU measurements, while the outputs are INS position errors. However, the uncertain nonlinear drift in MEMS inertial sensor measurement increases the nonlinear complexity of the inputs/outputs functional relationship being modeled. This will be a challenge to the nonlinear processing ability of ANN models. A generalized regression neural network (GRNN) has strong advantages in approximation capability compared with RBF neural network. This paper also demonstrates the potential of a GRNN in vehicle positioning.

In this paper, a highly reliable and cost-efficient vehicle positioning solution is proposed which integrates the information from MEMS-RISS, low-cost GPS, and supplementary sensors and sources. The RISS consists of one gyroscope and two accelerometers (1G2A), while the supplementary sensors and sources are composed of an electronic compass, a wheel speed sensor, and velocity constraints. Among the supplementary sensors and sources, the velocity information is used to estimate pitch and roll angle together with 1G2A. After eliminating the negative effect of uncertain nonlinear drift, the pitch and roll angle are used not only in INS mechanization but also as the inputs of GRNN module. Further, a novel distributed-dual-H∞ filtering (DDHF) mechanism is adopted to address the uncertain nonlinear drift and make full use of the supplementary sensors and sources. Meanwhile, the DDHF also provides training outputs for the GRNN module. Finally, the GRNN module with its distinguished ability to solve any function approximation problem is specially designed for the application of the MEMS-RISS. A hybrid methodology which combines part of the DDHF mechanism and the GRNN module is adopted to compensate for RISS position errors during GPS outages. The novel aspects of this paper can be summarized as follows:

(1) The uncertain nonlinear drift of MEMS inertial sensors is thoroughly considered in the application of MEMS-RISS. On one hand, an H∞ filter is employed to mitigate the effect of uncertain nonlinear drift in pitch and roll angle estimation. On the other hand, a DDHF mechanism is utilized for multi-sensor fusion. Due to the high robustness of the H∞ filter, the proposed methodology is essentially immune to the uncertain nonlinear drift of RISS.
(2) The hybrid methodology of error compensation has the advantages of both actual measurements and model predictions. Therefore, the proposed vehicle positioning solution can achieve highly reliable performance in the presence of GPS outages.

The organization of the paper is as follows: Section 2 gives an overview of the proposed positioning solution. Section 3 presents the estimation of pitch angle and roll angle based on the vehicle kinematic model and H∞ filter. Section 4 explains the detailed implementation of the DDHF mechanism. Section 5 describes the design of the GRNN module. Section 6 presents the results of experimental validation while Section 7 makes concluding remarks.

## 2. Overview of Proposed Solution

**Figure 1:**
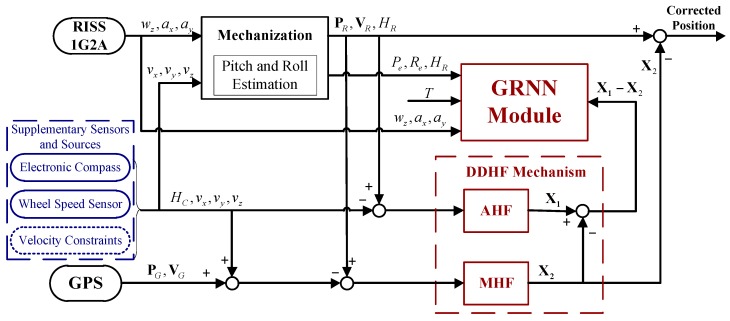
Diagram of the proposed positioning solution (learning mode).

**Figure 2:**
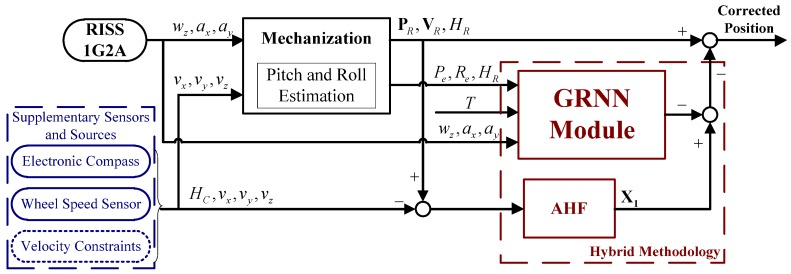
Diagram of the proposed positioning solution (prediction mode).

To achieve the goal of high reliability, a novel positioning solution is proposed by fusing multi-information from low-cost sensors and sources including low-cost GPS, MEMS-RISS, electronic compass, wheel speed sensor, and velocity constraints. The proposed positioning solution has two modes of operation, *i.e.*, the learning mode and prediction mode, which are dependent on the situation of GPS signal. For further clarification, the whole mechanism and functionality of the proposed positioning solution is illustrated in Figure 1 and Figure 2.

When GPS is available, the system operates in the learning mode, as shown in Figure 1. For the RISS, one single-axis gyroscope is mounted with its sensitive axis aligned with the vertical axis of the vehicle and measures the rotation rate of yaw angle in the body frame. Two accelerometers are mounted along the longitudinal and lateral axes of the vehicle, and the longitudinal and lateral accelerations of the vehicle are measured respectively. Also, supplementary sensors and sources are introduced in the proposed positioning system, *i.e.*, electronic compass, wheel speed sensor, and velocity constraints. The yaw angle is provided by the electronic compass, the longitudinal velocity is derived from the wheel speed sensor, and the lateral and vertical velocities are constrained under reasonable assumptions. Further, INS mechanization is processed by replacing the omitted gyroscopes and accelerometer with pseudo-signals, namely zero for gyroscopes and standard gravity for the vertical accelerometer. Meanwhile, pitch and roll angle are estimated based on the vehicle kinematic model and H∞ filter to eliminate the errors associated with position and velocity calculation in INS mechanization. Since the supplementary sensors and sources are inherently immune to GPS signal blockages, the measurement update associated with them can be executed during GPS outages. In order to make full use of the supplementary sensors and sources, a DDHF mechanism, which is composed of a main H∞ filter (MHF) and an auxiliary H∞ filter (AHF), is adopted for multi-sensor fusion. The GPS measurements, together with the observations provided by the supplementary sensors and sources, constitute the measurement vector of the MHF. As long as a GPS signal is available, the RISS position errors can be accurately estimated by the MHF. The AHF, which works in parallel with the MHF, only executes the measurement update associated with the supplementary sensors and sources. The differences between the state vectors of the two H∞ filters at each epoch are transferred to the GRNN module as the desired outputs. Meanwhile, the estimated pitch and roll angle, longitudinal velocity measured by wheel speed sensor, and the instantaneous time are fed to the GRNN as the corresponding inputs at the same epoch.

When the satellite signal is blocked in GPS-denied environments, the positioning system automatically switches to the prediction mode, as shown in Figure 2. Due to the unavailability of GPS measurements, the MHF is invalid and removed from the positioning system. However, the AHF can still be efficiently executed because its observations are immune to GPS outages. Meanwhile, with the current inputs, the trained GRNN module predicts the difference between the state vectors of the MHF and the AHF. Then, a hybrid methodology which combines the GRNN module with the always attainable AHF is utilized to estimate RISS position errors. Finally, the estimated position errors are removed from the corresponding RISS position components, and good positioning performance can be achieved by the proposed solution in GPS-denied environments.

## 3. Pitch and Roll Angle Estimation

In actual roadways, there are always certain pitch and roll angles of the vehicle caused by the road grade, the motion of the vehicle suspension, and *etc.*, which may be small, but their effect should not be neglected. Usually, pitch and roll angle are calculated by using the two accelerometers mounted along the longitudinal and lateral axes of the vehicle and the value for gravity at the present location [32]. Due to the uncertain nonlinear drift of MEMS accelerometers, one of the inadequacies of this method is that the pitch and roll angle are roughly calculated without filtering. The errors of pitch and roll angle will result in position and velocity errors in INS mechanization. Thus, to achieve better positioning accuracy, it is necessary to estimate the attitude angles using more information and eliminate the negative effect of uncertain nonlinear drift.

Using the kinematic relationship between the IMU output and the derivatives of the Euler angles, and assuming that the rotation rate of the Earth is negligible, the longitudinal and lateral motion of the vehicle can be modeled by the equations [33,34]

(1) $$\begin{array}{l} {\dot{v}}_{x}=a_{x}+w_{z}v_{y}-w_{y}v_{z}+g\sin P\\ {\dot{v}}_{y}=a_{y}-w_{z}v_{x}+w_{x}v_{z}-g\sin R\cos P \end{array}$$

where *w*_x_, *w*_y_, and *w*_z_ are the three angular velocities in the vehicle body frame. *v*_x_, *v*_y_, and *v*_z_ are the three linear velocities in the vehicle body frame, ${\dot{v}}_{x}$ and ${\dot{v}}_{y}$ are the differentiation of *v*_x_ and *v*_y_, respectively, *a*_x_ and *a*_y_ are the longitudinal and lateral accelerations in the vehicle body frame, *P* and *R* are pitch and roll angle, respectively, *g* denotes the acceleration due to gravity.

In common driving maneuvers, the vehicle does not sideslip or jump off the ground [35]. Thus, we can make the reasonable assumption that *v*_y_ ≈ 0, *v*_z_ ≈ 0 and ${\dot{v}}_{y}\approx 0$. Besides, when road surface is generally flat or changes smoothly, the changes in the pitch angles and roll angles of the vehicle are continuous and mild. Thus, it is reasonable to assume that *w*_x_ ≈ 0, *w*_y_ ≈ 0. Then, Equation (1) can be simplified as follows:

(2) $$\begin{array}{l} {\dot{v}}_{x}=a_{x}+g\sin P\\ 0=a_{y}-w_{z}v_{x}-g\sin R\cos P \end{array}$$

From Equation (2), the pitch and roll angle of the vehicle can be calculated if *w*_z_, *v*_x_, ${\dot{v}}_{x}$, *a*_x_, *a*_y_ and are available, *i.e.*,

(3) $$\begin{array}{l} P=\arcsin(\frac{{\dot{v}}_{x}-a_{x}}{g})\\ R=\arcsin(\frac{a_{y}-w_{z}v_{x}}{g\cos P}) \end{array}$$

Note that *v*_x_ is derived from the wheel speed sensor rather than the accelerometer. This is because when calculating velocity from accelerometer, any uncompensated accelerometer bias error will introduce an error in velocity during integration. The calculation of velocity from the wheel speed sensor avoids the integration, and thus a better performance can be achieved. Meanwhile, ${\dot{v}}_{x}$ at each time step can be calculated as:

(4) $${\dot{v}}_{x}\left(k\right)=\frac{v_{x}\left(k\right)-v_{x}\left(k-1\right)}{dt}$$

where $v_{x}(k)$ refers to the *v*_x_ at each time step. *dt* is the sample period. Since the output frequency of wheel speed sensor is 100 Hz, *dt* is 0.01 s here.

Besides, *w*_z_, *a*_x_, and *a*_y_ can be obtained from RISS. Since MEMS-RISS is utilized in this paper, the uncertain nonlinear drift of MEMS-RISS will cause large errors in pitch and roll angle calculations. As discussed in Section 1, the H∞ filter is essentially immune to uncertain nonlinear drift. Thus, it can be used to eliminate the negative effect of uncertain nonlinear drift in pitch and roll angle estimation.

The discretized state equation of H∞ filter can be presented as:

(5) $${\dot{X}}_{e}(k)=\Phi_{e}(k)\cdot X_{e}(k)+G_{e}(k)\cdot W_{e}(k)$$

where *k* is the discretized time step, $X_{e}={\left[\begin{array}{cc} P_{e} & R_{e} \end{array}\right]}^{T}$ is the state vector, *P*_e_ and *R*_e_ denote the estimation of pitch angle and roll angle, respectively, and **Ф**_e_ is the system state transition matrix. Since we assume that the pitch and roll angle at the current time step is the same as the next time step, we can get $\Phi_{e}=\left[\begin{array}{cc} 1 & 0\\ 0 & 1 \end{array}\right]$. **G**_e_ is the system noise input matrix, **W**_e_ is the system noise vector.

The discretized measurement equation can be expressed as:

**Z**_e_(*k*) = **H**_e_(*k*)**X**_e_(*k*) + **U**_e_(*k*) (6)

where **Z**_e_(*k*) is the observation vector, **H**_e_(*k*) is the observation matrix while **U**_e_(*k*) is the corresponding noise vector.

**H**_e_(*k*) is given as :

(7) $$H_{e}(k)=\left[\begin{array}{cc} 1 & 0\\ 0 & 1 \end{array}\right]$$

**Z**_e_(*k*) is defined as:

(8) $$Z_{e}(k)=\left[\begin{array}{c} P_{m}(k)\\ R_{m}(k) \end{array}\right]$$

where $P_{m}(k)$ and $R_{m}(k)$ denote the value of pitch and roll angle directly calculated by using Equation (3), respectively.

For the model described by the system state Equation (5) and the measurement Equation (6), we can execute the recursive procedure of the H∞ filter, which comprises three phases:

(1) Estimate the linear combination of state vector
  (9) $$Y_{e}(k)=L_{e}(k)X_{e}(k)$$
  where **Y**_e_(*k*) is the estimated vector and **L**_e_(*k*) is a user-defined matrix. Since we want to directly estimate **X**_e_(*k*), we set **L**_e_(*k*) = **I**. **I** is the identity matrix.
(2) Time propagation
  (10) $$X_{e}(k+1,k)=\Phi_{e}(k+1,k)X_{e}(k)$$

The recursion formula of **P**_e_(*k* + 1) is represented as

(11) $$\begin{array}{cc} P_{e}(k+1)= & \Phi_{e}(k+1,k)P_{e}(k)\Phi_{e}{\left(k+1,k\right)}^{T}+G_{e}(k)G_{e}{\left(k\right)}^{T}\\ & \;-\Phi_{e}(k+1,k)P_{e}(k)\left[\begin{array}{cc} H_{e}{\left(k\right)}^{T} & L_{e}{\left(k\right)}^{T} \end{array}\right]R_{e}{\left(k\right)}^{-1}\left[\begin{array}{c} H_{e}(k)\\ L_{e}(k) \end{array}\right]P_{e}(k)\Phi_{e}{\left(k+1,k\right)}^{T} \end{array}$$

with $R_{e}(k)=\left[\begin{array}{cc} I & 0\\ 0 & -\gamma^{2}I \end{array}\right]+\left[\begin{array}{c} H_{e}(k)\\ L_{e}(k) \end{array}\right]P_{e}(k)\left[\begin{array}{cc} H_{e}{\left(k\right)}^{T} & L_{e}{\left(k\right)}^{T} \end{array}\right]$, where γ is the specified performance bound.

(3) Measurement update
  (12) $$K_{e}(k+1)=P_{e}(k+1)H_{e}{\left(k+1\right)}^{T}{\left[I+H_{e}(k+1)P_{e}(k+1)H_{e}{\left(k+1\right)}^{T}\right]}^{-1}$$
  (13) $$X_{e}(k+1)=X_{e}(k+1,k)+K_{e}(k+1)[Z_{e}(k+1)-H_{e}(k+1)X_{e}(k+1,k)]$$

The value of the pitch and roll angle at each time step can be accurately estimated by executing the iterative procedure described by Equations (9)–(13). The estimated pitch and roll angle can be utilized to eliminate the errors associated with position and velocity calculation in INS mechanization. Besides, these angles can also act as inputs in the GRNN module.

## 4. DDHF Mechanism

Since the supplementary sensors and sources are immune to GPS outages, the measurement update associated with them can be executed to eliminate the corresponding accumulative errors of MEMS inertial sensors during GPS outages. However, the measurement update of observations cannot be sequentially processed in H∞ filter. Thus, a DDHF mechanism is adopted to overcome the drawback. Further, the adoption of DDHF promotes the hybrid methodology of error compensation during GPS outages. The DDHF mechanism comprises two filters, *i.e.*, AHF and MHF. The details of the mechanism are shown in the following paragraphs.

### 4.1. State Equation and Measurement Model

According to INS mechanization, the dynamic error model of the navigation parameters (*i.e.*, position, velocity, and attitude) can be described as [32,36]

(14) $$\begin{array}{l} \delta \dot{P}=D_{1}\delta P+D_{2}\delta V^{n}\\ \delta {\dot{V}}^{n}=f^{n}\times \psi^{n}-(2\delta \omega_{ie}^{n}+\delta \omega_{en}^{n})\times V^{n}-(2\omega_{ie}^{n}+\omega_{en}^{n})\times \delta V^{n}+C_{b}^{n}\delta f^{b}\\ {\dot{\psi }}^{n}=\delta \omega_{ie}^{n}+\delta \omega_{en}^{n}-(\omega_{ie}^{n}+\omega_{en}^{n})\times \psi^{n}-C_{b}^{n}\delta \omega_{ib}^{b} \end{array}$$

where $\delta P={\left[\begin{array}{ccc} \delta L & \delta \lambda & \delta h \end{array}\right]}^{T}$ is the position error vector (latitude, longitude, height), $\delta V^{n}={\left[\begin{array}{ccc} \delta V_{E} & \delta V_{N} & \delta V_{U} \end{array}\right]}^{T}$ is the velocity error vector (East, North, Up), $\psi^{n}={\left[\begin{array}{ccc} \psi_{E} & \psi_{N} & \psi_{U} \end{array}\right]}^{T}$ is the attitude error vector, and $V^{n}=\left[\begin{array}{ccc} V_{E} & V_{N} & V_{U} \end{array}\right]$ is the velocity vector in the navigation frame, **C**_b_^n^ is the ideal strapdown matrix, the definition of **C**_b_^n^ is given in Appendix, $\omega_{ie}^{n}$ is the angular rate vector of the rotation of the Earth relative to the inertial frame, $\omega_{en}^{n}$ is the angular rate vector of the rotation of the navigation frame relative to the Earth, $f^{n}$ is specific force in the navigation frame, $\delta f^{b}$ and $\delta \omega_{ib}^{b}$ are accelerometer biases and gyroscope drifts vectors in the body frame, respectively, and **D**_1_ and **D**_2_ are two 3 × 3 matrices whose non-zero elements are functions of the vehicle’s latitude and height.

In the proposed positioning system, the state vector of DDHF is composed of nine navigation parameter errors above (*i.e.*, position, velocity, and attitude), as follows:

(15) $$X={\left[\begin{array}{ccccccccc} \delta L & \delta \lambda & \delta h & \delta V_{E} & \delta V_{N} & \delta V_{U} & \psi_{E} & \psi_{N} & \psi_{U} \end{array}\right]}^{T}$$

Based on Equation (14) and the inertial sensor residual model [37], the discrete-time system state equation can be presented as

(16) X(k+1)=Φ(k+1,k)X(k)+G(k)W(k)

where **Ф**(*k* + 1, *k*) is the system state transition matrix, **G**(*k*) is the system noise input matrix, **W**(*k*) is the system noise vector.

The measurement equation can be expressed as

**Z**(*k*) = **H**(*k*)**X**(*k*) + **U**(*k*) (17)

where **Z**(*k*) is the measurement vector, **H**(*k*) is the observation matrix, and **U**(*k*) is the measurement noise vector.

According to Figure 1, the observations of DDHF comprise two parts, *i.e.*, observations provided by low-cost GPS and supplementary sensors and sources.

First, we will establish the measurement equation for velocity observations provided by wheel speed sensor and velocity constraints. Their measurements in the vehicle body frame have already described in Section 3, *i.e.*, *v*_x_, *v*_y_, *v*_z_. According to the coordinate transformation theory, we have

(18) $$\left[\begin{array}{c} v_{x}\\ v_{y}\\ v_{z} \end{array}\right]=C_{n}^{b}V^{n}-\left[\begin{array}{c} n_{x}\\ n_{y}\\ n_{z} \end{array}\right]$$

where **C**_n_^b^ is the transport matrix of **C**_b_^n^, the definition of **C**_n_^b^ is given in Appendix, $n_{x}$, $n_{y}$, and $n_{z}$ are the corresponding measurement noise for longitudinal, lateral, and vertical velocity, respectively.

Denoting the vehicle velocity vector calculated by RISS in the navigation frame as ${\hat{V}}^{n}={\left[\begin{array}{ccc} {\hat{V}}_{E} & {\hat{V}}_{N} & {\hat{V}}_{U} \end{array}\right]}^{T}$, and obviously ${\hat{V}}^{n}=V^{n}+\delta V^{n}$, the following equations can be yielded

(19) $$\left[\begin{array}{c} {\hat{V}}_{X_{b}}\\ {\hat{V}}_{Y_{b}}\\ {\hat{V}}_{Z_{b}} \end{array}\right]=C_{p}^{b}\cdot {\hat{V}}^{n}=C_{p}^{b}\cdot (V^{n}+\delta V^{n})$$

where ${\hat{V}}_{X_{b}}$, ${\hat{V}}_{Y_{b}}$, and ${\hat{V}}_{Z_{b}}$ represent the longitudinal, lateral, and vertical velocity calculated by RISS in the body frame, respectively, **C**_p_^b^ is the calculated strapdown matrix (the symbol p denotes the calculated platform frame).

Considering $C_{p}^{b}=C_{n}^{b}\left[I+\psi^{n}\times \right]$ and rearranging Equation (19), we can obtain

(20) $$\left[\begin{array}{c} {\hat{V}}_{X_{b}}\\ {\hat{V}}_{Y_{b}}\\ {\hat{V}}_{Z_{b}} \end{array}\right]=C_{n}^{b}(I+\psi^{n}\times )\left(V^{n}+\delta V^{n}\right)=C_{n}^{b}V^{n}+C_{n}^{b}\delta V^{n}+C_{n}^{b}\left(\psi^{n}\times \right)V^{n}+C_{n}^{b}\left(\psi^{n}\times \right)\delta V^{n}$$

Note that the last item of Equation (20) is a 2-order small quantity and can be neglected. Based on the skew-symmetric matrix features, we subtract Equation (18) from Equation (20) and then obtain

(21) $$\left[\begin{array}{c} \delta V_{X_{b}}\\ \begin{array}{l} \delta V_{Y_{b}}\\ \delta V_{Z_{b}} \end{array} \end{array}\right]=\left[\begin{array}{c} {\hat{V}}_{X_{b}}-v_{x}\\ \begin{array}{l} {\hat{V}}_{Y_{b}}-v_{y}\\ {\hat{V}}_{Z_{b}}-v_{z} \end{array} \end{array}\right]=C_{n}^{b}\delta V^{n}-C_{n}^{b}\left(V^{n}\times \right)\psi^{n}+\left[\begin{array}{c} n_{x}\\ \begin{array}{l} n_{y}\\ n_{z} \end{array} \end{array}\right]$$

where $V^{n}\times$ denotes the skew-symmetric matrix formed by the vector $V^{n}$.

Then, the measurement model about the observation provided by electronic compass can be built by subtracting the yaw angle measured by the electronic compass from the corresponding output of RISS. The measurement information and observation matrix are given in detail in [38].

Finally, the measurement model about GPS-aided RISS can be easily built by subtracting the GPS position and velocity measurements from the RISS’s corresponding outputs. The measurement information and observation matrix are given in detail in [36].

According to Figure 1, the AHF is about RISS aided by supplementary sensors and sources. The measurement equation is given as:

**Z**_1_ = **H**_1_**X**_1_ + **U**_1_ (22)

where **Z**_1_ is the observation vector of the AHF, **X**_1_ is the state vector of the AHF, **H**_1_ is the corresponding observation matrix, which can be determined by combing the measurement model about electronic compass-aided RISS and Equation (21), and **U**_1_ is the corresponding noise vector.

**Z**_1_ is given as: $Z_{1}={\left[\begin{array}{cccc} H_{R}-H_{C} & \delta V_{X_{b}} & \delta V_{Y_{b}} & \delta V_{Z_{b}} \end{array}\right]}^{T}$, where *H_R_* represents the yaw angle calculated by RISS, and *H_C_* represent the vehicle’s yaw angle measured by electronic compass.

The MHF is about RISS aided by GPS and supplementary sensors and sources. The measurement equation can be expressed as:

**Z**_2_ = **H**_2_**X**_2_ + **U**_2_ (23)

where **Z**_2_ is the observation vector of the MHF, **X**_2_ is the state vector of the MHF, **H**_2_ is the corresponding observation matrix, which can be determined by combing the measurement model about GPS-aided RISS, the measurement model about electronic compass-aided RISS, and Equation (21), and **U**_2_ is the corresponding noise vector.

**Z**_2_ is given as: $Z_{2}=\left[\begin{array}{cccccc} P_{R}-P_{G} & V_{R}-V_{G} & H_{R}-H_{C} & \delta V_{X_{b}} & \delta V_{Y_{b}} & \delta V_{Z_{b}} \end{array}\right]$, where **P**_R_ and **P**_G_ are the output position vector (latitude, longitude, and altitude) of RISS and GPS, respectively, and **V**_R_ and **V**_G_ are the output velocity vectors (East, North and Up) of RISS and GPS, respectively.

### 4.2. Implementation of DDHF

As mentioned above, the MHF and the AHF have the same state vector and state equation described by Equations (15) and (16) but have different measurement equations described by Equations (22) and (23), respectively. Thus, they execute a similar iterative procedure to those described by Equations (9)–(13), respectively.

When GPS is available, the state vector of the MHF **X**_2_ can easily be obtained and provides precise corrections to compensate for RISS errors. Meanwhile, the state vector of the AHF **X**_1_ is updated synchronously with **X**_2_. Their difference **X**_1_-**X**_2_ is the important training data of the GRNN module. Due to the centralized measurement update of GPS measurements and the observations provided by supplementary sensors and sources, the unavailability of GPS measurements leads to the invalidation of MHF during GPS outages. However, **X**_1_ can still update because the observations of AHF are immune to GPS signal blockages. The AHF ensures that the positioning system can still benefit from the measurement update associated with the supplementary sensors and sources during GPS outages. Besides, owing to the inherent properties of H∞ filter, the proposed positioning system is immune to the uncertain nonlinear drift. Thus, the beneficial effects of the DDHF are addressing the uncertain nonlinear drift of MEMS-RISS and making full use of the supplementary sensors and sources.

## 5. GRNN Module

The GRNN is a variation of an RBF neural network, which is based on nonparametric estimation commonly used in statistics [39]. It is a powerful regression tool that can solve any function approximation problem, including prediction, control, and general mapping [40,41].

For land vehicle applications, the horizontal positioning performance is generally the main concern, as presented in much of the literature [14,42] Besides, to simplify the GRNN network structure, the latitude and longitude components of the difference between two positioning error states associated with the MHF and the AHF are selected as the outputs of GRNN, *i.e.*,

**y** = [*pLpλ*] (24)

where *pL* denotes the latitude component of the difference between two positioning error states associated with the MHF and the AHF (the first element of **X**_1_-**X**_2_), while *pλ* is the corresponding longitude component (the second element of **X**_1_-**X**_2_).

When selecting the inputs, only the main factors affecting the outputs are considered. According to [8], the main sources of position errors are the inertial sensor measurements and attitude angles. Since we use RISS in this paper, the measurements of 1G2A, the estimated pitch and roll angle, and the yaw angle determined through INS mechanization are selected as the inputs. Besides, as presented in much literature [7,19], the instantaneous time is one of the most important inputs in ANN module. Therefore, the inputs of GRNN here can be chosen as

**x** = [*T w*_z_*a*_x_*a*_y_*P*_e_*R*_e_*H*_R_] (25)

where *T* denotes the time elapsed since the GPS signal is lost in prediction mode and denotes the time scale of the window in learning mode, *w*_z_ is the yaw rate measured by vertical gyroscope, *a*_x_ and *a*_y_ are the accelerations measured by longitudinal and lateral accelerometers, respectively, *P*_e_ and *R*_e_ are the estimated pitch and roll angles, respectively, and *H*_R_ is the yaw angle calculated by RISS.

**Figure 3:**
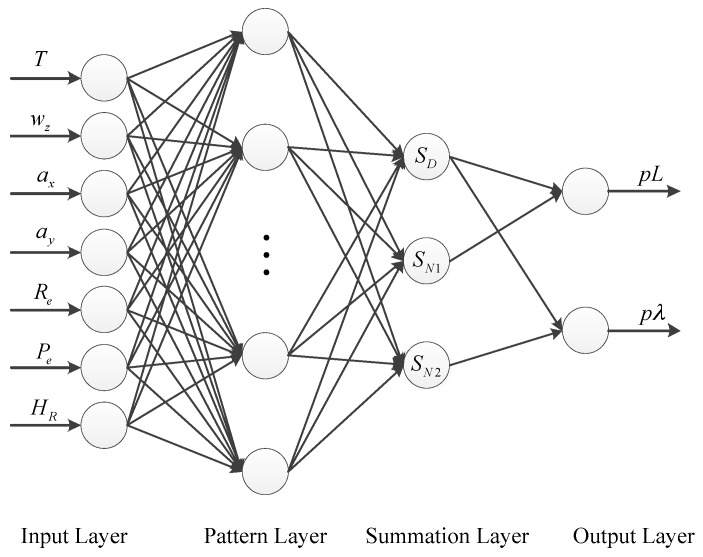
Schematic diagram of GRNN for the application in this paper.

The schematic diagram of the designed GRNN architecture is shown in Figure 3, which consists of four layers: the input layer, the pattern layer, the summation layer, and the output layer [43,44]. They are explained in detail as follows:

(1) Input Layer

The number of neurons in the input layer is equal to the dimension of inputs. The input layer is an allocation unit and it transfers inputs to the pattern layer.

(2) Pattern Layer

The number of neurons in the pattern layer is equal to the number of training samples *n*. Each neuron in the pattern layer represents a training pattern. The transfer function can be represented as:

(26) $$p_{i}=\exp[-\frac{{\left(x-x_{i}\right)}^{T}(x-x_{i})}{2\sigma^{2}}],i=1,2,\ldots n$$

where **x** is the current inputs, **x***_i_* is the *i*th input training sample, σ is the spread (or width) parameter, and its optimal value is determined via trial.

(3) Summation Layer

Each pattern layer unit is connected to the three neurons of the summation layer. The first summation neuron, also called the D-summation neuron, is used to calculate unweight outputs of the pattern neurons, *i.e.*,

(27) $$S_{D}=\sum_{i=1}^{n}p_{i}$$

The other two summation neurons are used to compute the sum of weighted responses of the pattern layer. These neurons are also called S-summation neurons. The mathematical equation can be expressed as:

(28) $$S_{Nj}=\sum_{i=1}^{n}w_{ij}p_{i},j=1,2$$

where *w_ij_* is the weighting between the pattern layer and the summation layer. The value of *w_ij_* is *j*th element of the *i*th output training sample.

(4) Output Layer

The number of neurons in the output layer is equal to the dimension of outputs. The output layer merely divides the output of each S-summation neuron by that of the d-summation neuron, yielding the predicted values of outputs y as

(29) $$y_{j}=\frac{S_{Nj}}{S_{D}},j=1,2$$

where *y_j_* is the *j*th element of **y**.

The designed GRNN is quite suitable for the application of RISS in the proposed positioning solution. When GPS is available, the model works in training mode. A non-overlapping sliding window with a window size of 50 is adopted to select training samples. The principal advantages are low computational load and timely updating of the GRNN parameters. When GPS signal is blocked, the trained GRNN model can predict the position errors with the reliable inputs. Then, accurate position corrections can be obtained by combining the predicted position errors and the outputs of AHF.

## 6. Experimental Validation

**Figure 4:**
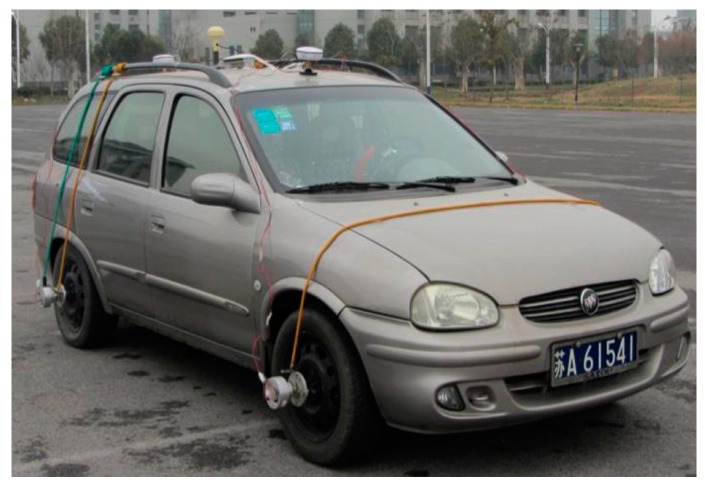
Test vehicle and its configuration.

To verify the positioning performance of the proposed solution in practice, experiments were conducted on a land vehicle. Sensor data were collected during the experiments, and the positioning solutions were run in post-processing using the logged data. The sensor data were recorded using a Buick Sail SRV vehicle, as shown in Figure 4. The test vehicle was equipped with low-cost NovAtel Superstar II GPS receiver (NovAtel, Calgary, Canada) with 1Hz update rate, MEMSIC MEMS-based IMU VG440CA-200 inertial sensors (MEMSIC, Wuxi, China) sampled at 100 Hz, KVH C100plus electronic compass (KVH, Middletown, CT, USA) with 1Hz update rate, and a wheel speed sensor based on a photoelectric encoder sampled at 100 Hz. The 1G2A of the RISS used in this research is the one vertical gyroscope and two horizontal accelerometers from the full six-degree-of-freedom (6-DoF) VG440CA-200. For the MEMS-based inertial sensors, the gyroscope has a bias stability of 10°/h and angle random walk of 4.5°/√h, while each accelerometer has bias stability of 1 mg and velocity random walk of 1 m/s/√h. The accuracies of other sensors (1 σ) are 0.05 m/s and 3 m for the velocity and position with available GPS signal, 0.5° for the yaw angle of electronic compass, and 0.05 m/s for the longitudinal velocity of wheel speed sensor. The electronic compass is mounted on an aluminum rod to avoid magnetic interference and it is carefully calibrated before use. Moreover, an accurate and reliable NovAtel SPAN-CPT system was used as a reference for quantitative comparison. The positioning accuracy of SPAN-CPT system was 0.01 m with GNSS observations and 0.02 m during 10 s outages, while the attitude accuracy was 0.015° for both pitch angle and roll angle.

Several road-test trajectories were carried out using the setup described above. The trajectories presented here show the performance in environments encompassing different conditions. One of the trajectories carried out in the suburb area with several stops due to traffic lights. This trajectory was an open field where a sufficient number of satellites were available for both low-cost GPS and SPAN-CPT system throughout the whole procedure of the experiment. Simulated GPS outages were introduced in this trajectory. The other trajectory was carried out in a typical urban scenario with frequent driving maneuvers such as lane-changes and sudden accelerations/decelerations. Also, a real GPS-denied environment was included in this trajectory.

### 6.1. Test 1: Performance of the H∞ Filter for Pitch and Roll Angle Estimation

The performance of pitch and roll angle estimation based on the H∞ Filter is evaluated in this test. Since pitch and roll angle estimation is part of the whole positioning system, it can also be verified when the overall performance of the proposed system is evaluated. Thus, we only demonstrate the estimation results in trajectory 1 here.

**Figure 5:**
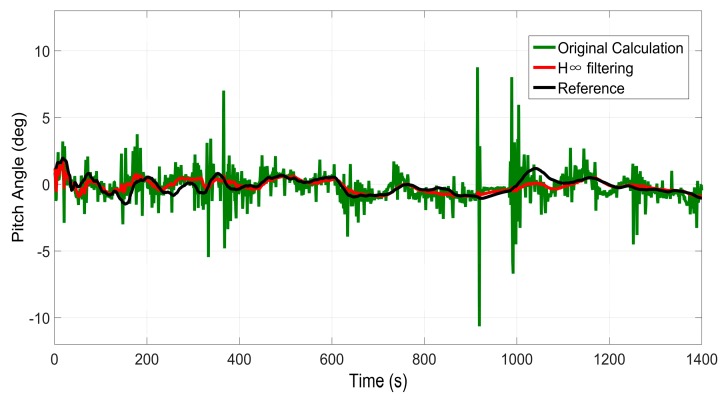
Results of pitch angle estimation in trajectory 1.

**Figure 6:**
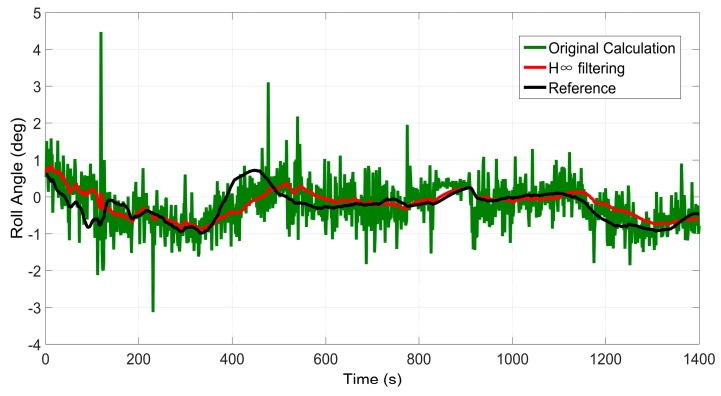
Results of roll angle estimation in trajectory 1.

Figure 5 and Figure 6 display the results of pitch angles and roll angles computed by the two approaches, *i.e.*, the original calculation approach and H∞ filtering approach, respectively. The original calculation approach means that the roll angles and pitch angles are computed according to Equation (3), directly utilizing the data obtained from the inertial sensors. The H∞ filtering approach means that the roll angles and pitch angles are estimated using the method presented in Section 3. Meanwhile, the corresponding measurements output by SPAN-CPT are used as the references. It can be seen from Figure 5 and Figure 6 that the angles estimated by the H∞ filtering approach are much smoother than those calculated by the original calculation approach. Due to the uncertain nonlinear drift of MEMS inertial sensors, there is much noise in the originally calculated pith and roll angle. Besides, the accuracy of the H∞ filtering approach is obviously better than that of the original calculation approach.

Table 1 gives the error statistics of these angles computed by the two approaches. It can be determined that the STD value is 62% smaller after H∞ filtering for pitch angle, while the magnitude is 45% for roll angle.

In conclusion, the proposed H∞ filtering approach can significantly reduce the noise and produce a reliable estimation of the pitch and roll angle, which will be beneficial for the subsequent procedures, including INS mechanization and GRNN module.

### 6.2. Test 2: Performance Evaluation of the Proposed Positioning Solution in Trajectory 1

**Figure 7:**
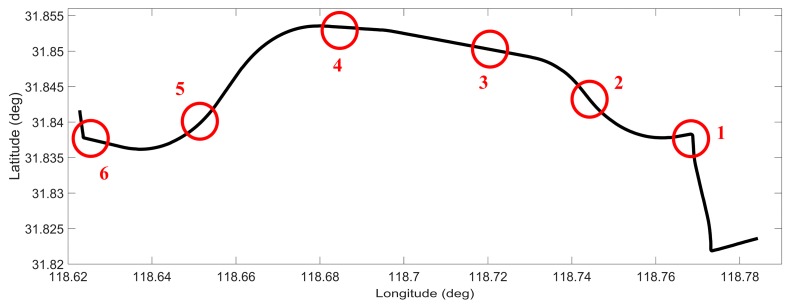
Road-test trajectory 1 with simulated GPS outages indicated.

In this test, the overall performance of the proposed positioning solution was evaluated in trajectory 1. This road-test trajectory was conducted in the suburb of Nanjing, China and the number of accessible satellites was greater than five during the whole test. The road test was performed for nearly 30 min of continuous vehicle navigation and a distance of around 17 km. Typical driving maneuvers such as turns and stops at the traffic lights were conducted during the test. Six simulated 50 s GPS signal outages were intentionally introduced at different locations along the trajectory, as shown in Figure 7. Different dynamics and motion types (straight, curve and 90° turn) were considered when choosing the simulated GPS outages.

Note that the position errors in the following denote the horizontal Euclidean distance error between the estimated position and the corresponding reference, which is the main concern for land vehicle positioning. The proposed solution (GRNN-DDHF) was compared with four different solutions: the hybrid solution which combines DDHF with RBF neural network (RBF-DDHF), DDHF solution without ANN compensation (DDHF), KF solution without ANN compensation (KF), and KF solution with general method (KF-GENERAL). The general method meant the INS mechanization was performed by simply replacing omitted inertial sensors with pseudo-signals and the pitch and roll angle were calculated without filtering. Except for the KF-GENERAL solution, pitch and roll angle were all estimated in the other four solutions using the method presented in Section 3. It is also worthwhile to mention here that the KF solution used the same system model as DDHF, *i.e.*, the INS dynamic error model described in Equation (14). The difference was that there were twelve elements in the state vector of KF. Apart from the nine elements in the state vector of DDHF, two accelerometer biases and one gyroscope drift were also included in the state vector of KF to estimate the corresponding sensor errors. Besides, the KF used first-order Gauss Markov models for the stochastic errors of inertial sensors. Further, the mechanism of KF solution was similar to that of DDHF, *i.e.*, the two H∞ filters in DDHF were replaced by KF with the same observations respectively. According to the majority of publications in the same field, the RBF neural network has been widely regarded as the most remarkable ANN in the past few decades [28,29,30]. Thus, it is chosen as a comparison of GRNN in this paper. The RBF module was designed with the same inputs and outputs as GRNN. Besides, the same 50 s non-overlapping sliding window was utilized to train the RBF module. The learning procedure continued as long as the GPS signals were available, whereas in case of outages, the trained RBF and GRNN module were utilized to predict and compensate for the RISS position errors. In the absence of GPS outages, all five solutions provided accurate position information with GPS measurements. Therefore, we focused on the comparison of the performances during GPS outages among the five solutions.

Table 2 and Table 3 give a quantitative comparison of the maximum and RMS position errors among the five solutions during the six GPS outages in trajectory 1, respectively. It can be seen that the solutions with ANN compensation, *i.e.*, GRNN-DDHF and RBF-DDHF, can achieve much lower position errors than the other three solutions, because ANNs can mimic the latest RISS sensor errors and remove them from the corresponding RISS position components, thereby improving the positioning accuracy. In addition, due to the better approximation capability, the maximum error of the proposed GRNN-DDHF solution is 36% lower than that of the RBF-DDHF solution on average. When it comes to the RMS error, the proposed solution achieves a 39% lower value than RBF-DDHF-RISS solution.

The position error results also show that the KF solution outperforms the KF-GENERAL solution, because the omitted gyroscopes were simply replaced by pseudo-signals in the general method and the calculations of pitch and roll angle were not correct, thus resulting in large position errors. Conversely, the pitch and roll angle were accurately estimated in KF solution. From Table 2 and Table 3, KF solution achieves a 3% performance improvement over KF-GENERAL solution. Furthermore, the results show that the positioning system is better when using DDHF than KF. The reason for this is that DDHF has the ability to deal with the uncertain nonlinear drift of MEMS inertial sensors. DDHF does not require the statistical properties of inertial sensor errors, while KF needs predefined accurate models for inertial sensor errors. In other words, DDHF is essentially immune to the uncertain nonlinear drift of MEMS-RISS with high robustness. From Table 2 and Table 3, the DDHF solution achieves a 20% performance improvement over the KF solution.

Through examining Table 2 and Table 3 carefully, it can be seen that the position error during outage 1 in the proposed solution is lower than that during outage 6. Although they both involved the 90° turn motion type, the vehicle speed during outage 1 was lower than 25 km/h, while it was nearly 40 km/h during outage 6. That is to say, the driving maneuvers change slightly during outage 1, which coincides with the assumptions of velocity constraints. Consequently, velocity constraints are more efficient in general driving conditions.

**Figure 8:**
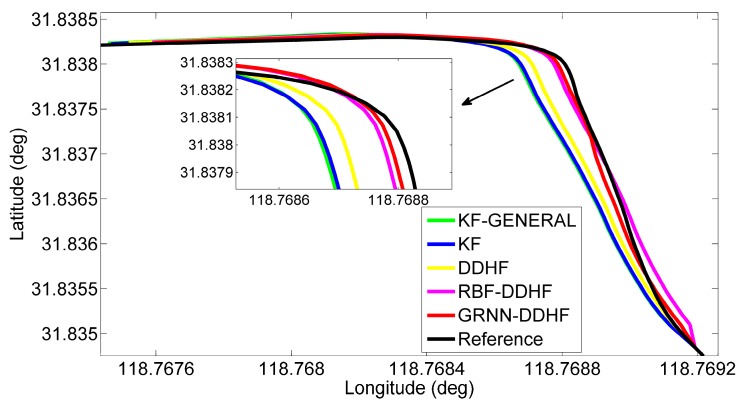
Positioning results during GPS outage 1 in trajectory 1.

**Figure 9:**
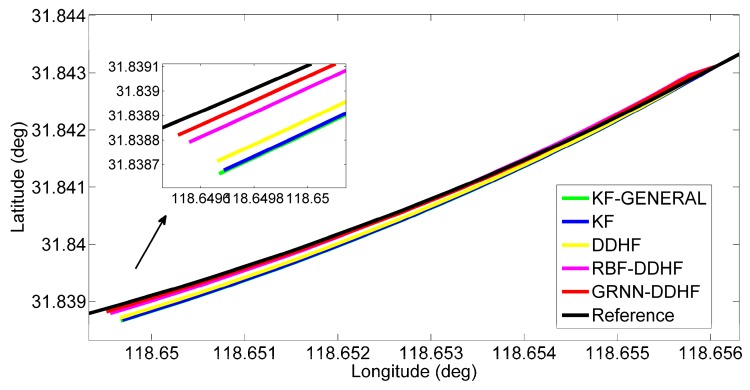
Positioning results during GPS outage 5 in trajectory 1.

To show the details of the performance during some of these outages, two representative simulation outages, *i.e.*, 1 and 5, were chosen to show the trajectories output by five solutions, as illustrated in Figure 8 and Figure 9.

Figure 8 illustrates the positioning results for outage 1, which is introduced in a 90° turn. The proposed solution predicted a position component (in red) that possesses less drift in comparison to the other four solutions. Moreover, the maximum position error is found to improve by 24% over the RBF-DDHF solution, 67% over the DDHF solution, 75% over the KF solution, and 77% over the KF-GENERAL solution. When examining the RMS errors, the percentage improvement is found to improve by 16% over the RBF-DDHF solution, 71% over the DDHF solution, 78% over the KF solution and 80% over the KF-GENERAL solution. Although the RBF-DDHF solution is a little bit better than the proposed solution during the turn portion, it has an evident position drift at the beginning of the outage. Thus, the proposed GRNN-DDHF solution is the best overall.

Figure 9 gives the positioning results for outage 5, which corresponds to the portion of the trajectory for vehicle motion along a curve. The trajectory predicted by the proposed solution is quite close to the reference trajectory (in black) and thus effectively reduces the maximum position error by 36% compared to the RBF-DDHF solution, 71% compared to the DDHF solution, 76% compared to the KF solution, and 76% compared to the KF-GENERAL solution, and the percentage improvement in RMS position error is found to be 40%, 77%, 80%, and 81%, respectively.

### 6.3. Test 3: Further Evaluation of the Reliability of the Proposed Positioning Solution

**Figure 10:**
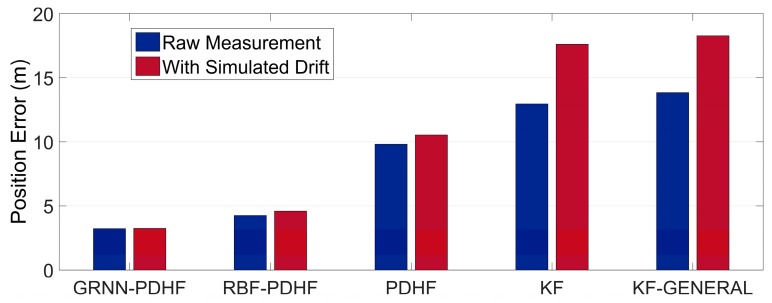
Maximum position error during outage 1 in trajectory 1 while using raw measurement and with extra simulated uncertain nonlinear drift.

**Figure 11:**
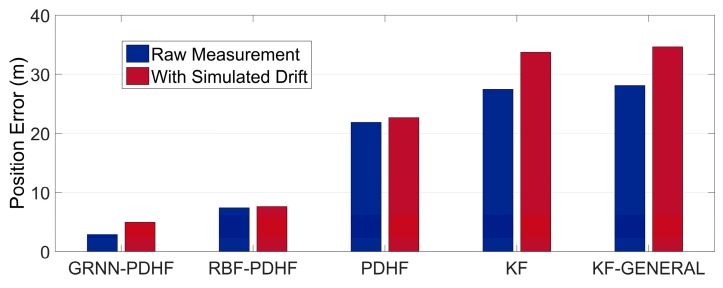
Maximum position error during outage 3 in trajectory 1 while using raw measurement and with extra simulated uncertain nonlinear drift.

**Figure 12:**
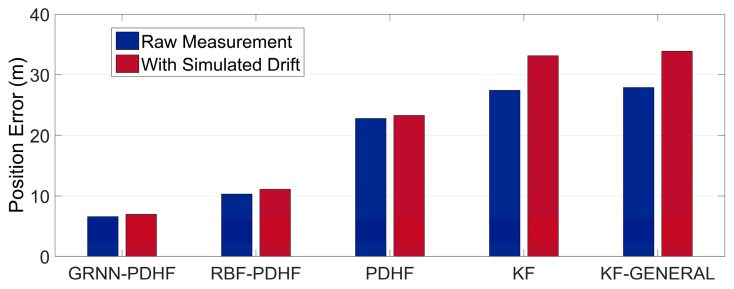
Maximum position error during outage 5 in trajectory 1 while using raw measurement and with extra simulated uncertain nonlinear drift.

In order to further test the reliability of the proposed solution, we inserted biases in the measurements of the 1G2A during the periods of simulated GPS outages. For convenience and effectiveness, we chose constant biases to simulate uncertain nonlinear drift, *i.e.*, bias of 100 mg into the two accelerometers and bias of 300°/h into the gyroscope. After inserting biases, the statistical properties of inertial sensor errors were dramatically changed during simulated outages, which was unknown to the KF. The real uncertain nonlinear drift could cause the same problem. The wrong error model would cause rapid degradation in KF performance. However, DDHF was envisioned to be less sensitive to the changes of statistical properties. Table 4 and Table 5 show the results of maximum and RMS position errors among the five solutions during the six GPS outages in trajectory 1 with extra simulated uncertain nonlinear drift. As expected, comparing Table 2 and Table 4, it can be determined that, after the statistical properties of INS sensor errors are intentionally changed, the maximum position error of solutions with DDHF only increases 9% on average while the increase of those with KF is 24%. Meanwhile, comparing Table 3 and Table 5, it can be found that the increase of RMS position error of solutions with DDHF is also much lower than it with KF. It can be concluded that the modeling errors caused by uncertain nonlinear drift of MEMS inertial sensors have a great effect on the performance of KFs, including any form of KF which needs predefined sensor error models. In contrast, DDHF can maintain good performance facing the same situation. In order to clearly demonstrate the reliability of the proposed solution, we chose three outages, *i.e.*, 1, 3, and 5, to show the comparisons of the maximum position errors before and after inserting biases among the five solutions, as depicted in Figure 10, Figure 11 and Figure 12.

### 6.4. Test 4: Performance Evaluation of the Proposed Positioning Solution in Trajectory 2

**Figure 13:**
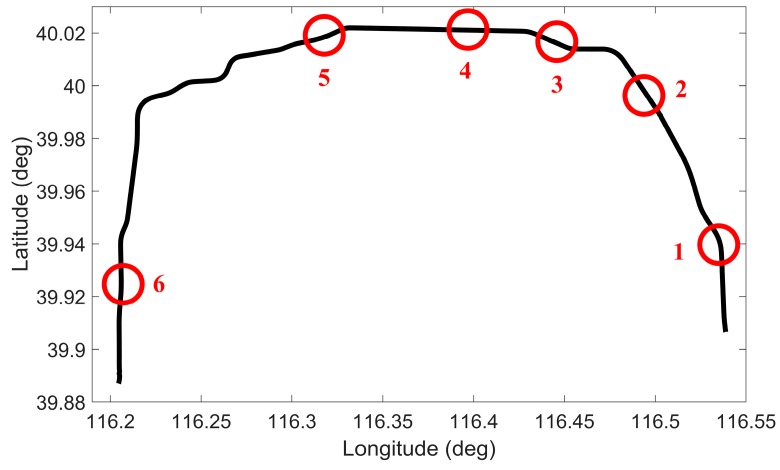
Road-test trajectory 2 with GPS outages indicated.

To further validate the performance of the proposed solution, trajectory 2 in urban area with real GPS outages was used for this purpose. This trajectory was carried out on the Fifth Ring Road in Beijing, China. The road test was performed for nearly 40 min and a distance of around 48 km. Although there were no traffic lights on the Fifth Ring Road, frequent driving maneuvers such as lane-changes and sudden accelerations/decelerations were conducted due to the typical crowded urban road. During the whole test, real GPS outages were caused by overpasses, trees, and road infrastructure. Since some periods of real GPS outages were shorter than 50 s, the selected outages were all extended to 50 s for convenient comparison. Straight portions and curves were also considered when selecting outages in this trajectory, as shown in Figure 13.

Table 6 and Table 7 show the maximum and RMS position errors during the outages in trajectory 2 for the five solutions, respectively. Due to the frequent changes of driving maneuvers on urban roadways, it is obvious that the position errors achieved by each solution are larger than those in trajectory 1 on average. However, the proposed GRNN-DDHF solution still has the best maximum and RMS position errors.

The results from Table 6 and Table 7 confirm the results of the previous trajectory. Comparing the GRNN-DDHF solution and RBF-DDHF solution with the other three solutions, the superiority of ANN models is clear. Again, the KF solution outperforms the KF-GENERAL solution because of the estimation of pitch and roll angle rather than without. Comparing the DDHF solution with the KF solution, the general advantages of the H∞ filter are further validated.

**Figure 14:**
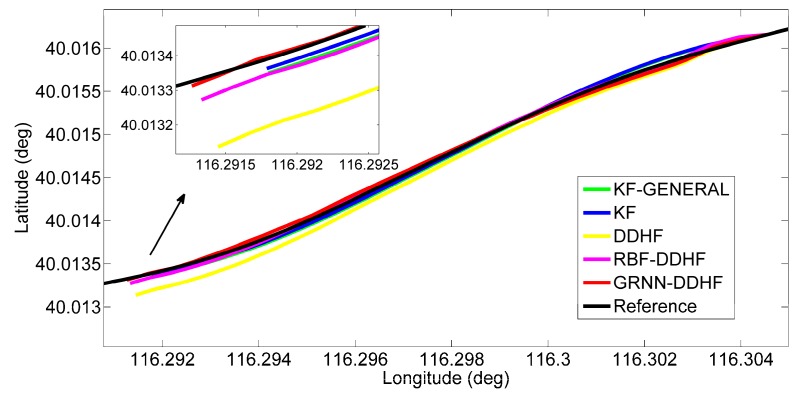
Positioning results during GPS outage 5 in trajectory 2.

One representative outage, *i.e.*, 5, was chosen to show the trajectories output by five solutions, as illustrated in Figure 14. Outage 5 belonged to a typical curve road. It can be clearly seen that the KF solution and KF-GENERAL solution have large position drift in the longitudinal direction, while DDHF solution has large position drift in the latitudinal direction. However, the proposed solution and RBF-DDHF solution are able to provide better positioning accuracy than the other three solutions. The maximum position error is found to improve by 50% compared to the RBF-DDHF-RISS solution, 77% compared to the DDHF solution, 79% compared to the KF solution and 79% compared to the KF-GENERAL solution, and the RMS position error is found to improve by 70%, 80%, 80%, and 82%, respectively.

## 7. Conclusions

This paper has presented a cost-efficient solution for land vehicle positioning, which integrates low-cost GPS, MEMS-RISS (1G2A), an electronic compass, a wheel speed sensor, and velocity constraints.

The proposed solution benefits from the advantages of the H∞ filter and GRNN to achieve a reliable positioning solution. First, H∞ filtering has been presented to obtain more accurate and reliable pitch and roll information. Further, the DDHF mechanism is adopted to fuse the data of low-cost GPS, MEMS-RISS, and supplementary sensors and sources. Finally, the GRNN module is designed with the inputs of the instantaneous time, the measurements of 1G2A, the estimated pitch and roll angle, and the yaw angle calculated by the RISS, while the outputs of the GRNN module are the differences between the state vectors of the MHF and the AHF. When GPS is available, DDHF provides accurate corrections for RISS positions. In cases of GPS outage, the GRNN module compensates for the RISS position errors together with the outputs of DDHF. Consequently, the proposed solution is able to achieve accurate and reliable positioning performance in GPS-denied environments.

The proposed positioning solution has been successfully implemented and tested with real road-test trajectories. Through comparison with the other four representative positioning solutions, it can be concluded that the research fulfills the basic aim of proposing a highly reliable and cost-efficient vehicle positioning solution.

It is worthwhile to mention here that wheel speed sensors and some inertial sensors have already been installed in a large number of vehicles and their information can be directly read via the vehicle controller area network (CAN) bus. Further research will combine more information from the vehicle CAN bus to further increase positioning accuracy with a lower cost.

## Figures and Tables

**Table 1:** Statistics of Angle Errors.

Error Item	Original Calculation		H∞ Filtering	
	Mean	STD	Mean	STD
Pitch Error (°)	0.0507	1.0197	0.0246	0.3907
Roll Error (°)	0.0336	0.5601	0.0578	0.3085

**Table 2:** Maximum position errors during GPS outages for Trajectory 1.

Outage Num.	Maximum Error (m)				
	GRNN-DDHF	RBF-DDHF	DDHF	KF	KF-GENERAL
1	3.21	4.25	9.80	12.95	13.83
2	7.36	10.84	20.36	26.76	27.42
3	2.91	7.41	21.85	27.47	28.11
4	10.13	19.15	21.92	27.82	28.49
5	6.58	10.32	22.78	27.43	27.89
6	12.57	14.45	20.36	25.44	25.65

**Table 3:** RMS position errors during GPS Outages for Trajectory 1.

Outage Num.	RMS Error (m)				
	GRNN-DDHF	RBF-DDHF	DDHF	KF	KF-GENERAL
1	0.78	0.93	2.71	3.54	3.82
2	1.38	3.19	5.84	7.50	7.69
3	0.65	2.07	6.30	7.74	7.92
4	2.94	4.60	6.45	7.90	8.10
5	1.51	2.53	6.56	7.75	7.88
6	2.98	3.45	6.14	7.45	7.55

**Table 4:** Maximum position errors during GPS outages for Trajectory 1 with extra simulated uncertain nonlinear drift.

Outage Num.	Maximum Error (m)				
	GRNN-DDHF	RBF-DDHF	DDHF	KF	KF-GENERAL
1	3.24	4.60	10.54	17.61	18.26
2	10.64	11.49	20.41	33.34	34.10
3	4.95	7.62	22.64	33.71	34.61
4	10.69	19.54	22.84	34.04	34.98
5	6.97	11.09	23.29	33.13	33.88
6	13.14	14.72	20.53	29.81	30.70

**Table 5:** RMS position errors during GPS outages for Trajectory 1 with extra simulated uncertain nonlinear drift.

Outage Num.	RMS Error (m)				
	GRNN-DDHF	RBF-DDHF	DDHF	KF	KF-GENERAL
1	0.72	1.10	2.01	5.23	5.49
2	2.10	3.14	5.43	9.50	9.71
3	0.64	2.22	5.80	9.61	9.86
4	2.58	4.53	6.05	9.76	10.02
5	1.54	2.95	6.64	9.44	9.63
6	2.94	3.57	5.77	8.91	9.29

**Table 6:** Maximum position errors during GPS outages for Trajectory 2.

Outage Num.	Maximum Error (m)				
	GRNN-DDHF	RBF-DDHF	DDHF	KF	KF-GENERAL
1	11.63	17.15	21.78	22.94	25.08
2	7.15	13.52	19.89	27.23	27.42
3	15.20	16.02	25.82	28.13	28.53
4	6.29	10.24	25.78	29.39	30.17
5	6.74	13.41	29.99	31.55	32.44
6	12.49	13.51	19.33	23.16	23.23

**Table 7:** RMS position errors during GPS outages for Trajectory 2.

Outage Num.	RMS Error (m)				
	GRNN-DDHF	RBF-DDHF	DDHF	KF	KF-GENERAL
1	2.23	3.78	5.81	6.57	7.07
2	1.35	3.22	4.15	6.28	6.49
3	2.56	4.02	6.59	7.55	7.74
4	1.51	2.86	4.75	5.28	5.51
5	1.13	3.73	5.75	5.77	6.22
6	2.20	2.54	4.23	5.46	5.46

## Abbreviations

The following abbreviations are used in this manuscript:

DDHF	distributed-dual-H∞ filtering
MHF	main H∞ filter
AHF	auxiliary H∞ filter
GRNN	generalized regression neural network
GPS	Global Positioning System
INS	Inertial Navigation System
KF	Kalman filter
MEMS	micro-electro-mechanical system
IMU	inertial measurement unit
RISS	reduced inertial sensor system
ANN	artificial neural network
RBF	Radial Basis Function
ANFIS	Adaptive Neuron-Fuzzy Inference System
1G2A	one gyroscope and two accelerometers
CAN	Controller Area Network

## Appendix

$$C_{b}^{n}=\left[\begin{array}{ccc} \cos H_{t}\cos P_{t} & \cos H_{t}\sin P_{t}\sin R_{t}-\sin H_{t}\cos R_{t} & \cos H_{t}\sin P_{t}\cos R_{t}+\sin H_{t}\sin R_{t}\\ \sin H_{t}\cos P_{t} & \sin H_{t}\sin P_{t}\sin R_{t}+\cos H_{t}\cos R_{t} & \sin H_{t}\sin P_{t}\cos R_{t}-\cos H_{t}\sin R_{t}\\ -\sin P_{t} & \cos P_{t}\sin R_{t} & \cos P_{t}\cos R_{t} \end{array}\right]$$

where *H*_t_, *P*_t_, and *R*_t_ are true yaw, pitch, and roll angles of the vehicle, respectively.

$$C_{n}^{b}=\left[\begin{array}{ccc} \cos H_{t}\cos P_{t} & \sin H_{t}\cos P_{t} & -\sin P_{t}\\ \cos H_{t}\sin P_{t}\sin R_{t}-\sin H_{t}\cos R_{t} & \sin H_{t}\sin P_{t}\sin R_{t}+\cos H_{t}\cos R_{t} & \cos P_{t}\sin R_{t}\\ \cos H_{t}\sin P_{t}\cos R_{t}+\sin H_{t}\sin R_{t} & \sin H_{t}\sin P_{t}\cos R_{t}-\cos H_{t}\sin R_{t} & \cos P_{t}\cos R_{t} \end{array}\right]$$
